# Psychological distress in an obese sample of 12-15 years old adolescents

**DOI:** 10.1192/j.eurpsy.2023.1572

**Published:** 2023-07-19

**Authors:** V. Lama, A. Ferizi

**Affiliations:** European University of Tirana, Tirana, Albania

## Abstract

**Introduction:**

The prevalence of overweight and obesity in youths has increased considerably and is related to several health and psychological issues. According to a study from Public Health Institute in Albania 7.7% of children are obese (Hyska et al., Albania Public Health Institute 2013), but there are no data for adolescents.

**Objectives:**

Recently a special attention is given to psychological consequences of obesity in youths. The aim of this study was to explore body image, self-esteem and psychosocial coping of obese adolescents.

**Methods:**

The sample consisted of 200 obese Albanian adolescents aged 12-15 years old, 134 females and 66 males. BMI was considered as the obesity index. We used WHO growth references to determine body mass index (BMI) percentiles. Participants were students enrolled in grade 6 to 9. We used Rosenberg self-esteem scale and Body Shape Questionnaire by Cooper et al. which measures recent concerns about appearance feelings and is a widely used self-report measure of body shape dissatisfaction. Also, a self-constructed questionnaire related to social interactions, stigma and psychosocial coping was distributed to adolescents.

**Results:**

We found out an association between obesity in youths and self-esteem (77% of the sample had a low self-esteem), body image (72% reported moderate and strong concerns for their body appearance), as well as problems of social interaction. Low self-esteem was more prevalent in females (53%) compared to males (20%). There was no significant gender difference regarding body appearance concerns (p >0.05). The majority of the sample (64%) reported to be bullied, but they did not tell anyone about it.

**Conclusions:**

Our findings reinforce the negative effects of obesity in early adolescence. Body image is closely related to self-esteem and this is noticeably manifested during early adolescence. Low self-esteem might be related with body shape dissatisfaction, but we didn‘t further explore the correlation. This sample of obese adolescents reported to be largely exposed to bullying (64%), still it is of great concern that none of the adolescents did confess it to family, teachers, school psychologist or friends.

**Disclosure of Interest:**

None Declared

